# mRNA-LNP expressing PfCSP and Pfs25 vaccine candidates targeting infection and transmission of *Plasmodium falciparum*

**DOI:** 10.1038/s41541-022-00577-8

**Published:** 2022-12-01

**Authors:** Clifford T. H. Hayashi, Yi Cao, Leor C. Clark, Abhai K. Tripathi, Fidel Zavala, Garima Dwivedi, James Knox, Mohamad-Gabriel Alameh, Paulo J. C. Lin, Ying K. Tam, Drew Weissman, Nirbhay Kumar

**Affiliations:** 1 Department of Global Health, Milken Institute School of Public Health, George Washington University, Washington, DC 20052 USA; 2 Johns Hopkins Malaria Research Institute, Department of Molecular Microbiology and Immunology, Bloomberg School of Public Health, Johns Hopkins University, Baltimore, MD 21215 USA; 3 Department of Medicine, University of Pennsylvania, Philadelphia, PA 19104 USA; 4 Department of Pathology, University of Pennsylvania, Philadelphia, PA 19104 USA; 5 Acuitas Therapeutics, Vancouver, BC Canada

**Keywords:** RNA vaccines, Malaria

## Abstract

Malaria is a deadly disease responsible for between 550,000 and 627,000 deaths annually. There is a pressing need to develop vaccines focused on malaria elimination. The complex lifecycle of *Plasmodium falciparum* provides opportunities not only to target the infectious sporozoite stage, introduced by anopheline mosquitoes, but also the sexual stages, which are ingested by mosquitoes during blood feeding, leading to parasite transmission. It is widely recognized that a vaccine targeting multiple stages would induce efficacious transmission reducing immunity. Technological advancements offer new vaccine platforms, such as mRNA-LNPs, which can be used to develop highly effective malarial vaccines. We evaluated the immunogenicity of two leading *P. falciparum* vaccine candidates, Pfs25 and PfCSP, delivered as mRNA-LNP vaccines. Both vaccines induced extremely potent immune responses when administered alone or in combination, which were superior to Pfs25 and PfCSP DNA vaccine formulations. Purified IgGs from Pfs25 mRNA-LNPs immunized mice were highly potent in reducing malaria transmission to mosquitoes. Additionally, mice after three and four immunizations with PfCSP mRNA-LNP provided evidence for varying degrees of protection against sporozoite challenge. The comparison of immune responses and stage-specific functional activity induced by each mRNA-LNP vaccine, administered alone or in combination, also supports the development of an effective combination vaccine without any risk of immune interference for targeting malaria parasites at various life cycle stages. A combination of vaccines targeting both the infective stage and sexual/midgut stages is expected to interrupt malaria transmission, which is critical for achieving elimination goals.

## Introduction

Malaria is caused by *Plasmodium* parasites transmitted by female anopheline mosquitoes. As of 2020, malaria was prevalent in greater than 90 countries accounting for 241 million cases with an estimated 627,000 deaths^1,2^. Over the past few decades, progress has been made toward reducing malaria incidence through mosquito control interventions and increased access to antimalarial drugs. Unfortunately, drug resistance towards frontline antimalarial drugs continues to increase, and overall progress in incidence reduction has started to stagnate^2,3^. Therefore, interventions, such as vaccines, are needed to achieve further progress toward malaria elimination. There has been progress in vaccine development, where the RTS,S/AS01 vaccine became the first and only approved vaccine to combat the disease^2^. However, the RTS,S/AS01 vaccine is only partially effective in protecting against clinical malaria with the efficacy waning over multiple years^4,5^. Therefore, next-generation vaccines will need to apply new strategies for improved efficacy, and possibly target parasites at multiple life cycle stages.

Due to the complex parasite life cycle, there are three distinct types of vaccines in development: pre-erythrocytic, blood-stage, and transmission-blocking vaccines. Pre-erythrocytic vaccines target sporozoite and liver-stage parasites with the aim of eliciting immune responses to prevent infection. In contrast, blood-stage vaccines target the disease-causing parasites to elicit an immune response to limit parasite burden, thereby reducing disease severity. On the other hand, transmission-blocking vaccines target the sexual stage parasites in the female mosquitoes, leading to the disruption of the sexual life cycle and cessation of parasite development, and reduction of transmission. It is widely accepted that a vaccine comprised of multiple antigen combinations and targeting multiple stages will likely produce a highly effective vaccine, to stop malaria transmission^6–9^.

One of the primary targets for pre-erythrocytic vaccine development is the *Plasmodium falciparum* circumsporozoite protein (PfCSP). PfCSP is a protein containing three regions; a N terminal domain, an immunodominant repeat region and a C terminal domain which contains multiple T cell epitopes^10^. PfCSP is expressed on the infectious sporozoite that contributes to parasite motility and hepatocyte invasion^11^. Numerous vaccine platforms, such as virus-like particles (VLPs), nanoparticles, live vectors, and DNA plasmids, have been evaluated for PfCSP vaccine development with mixed success^6,12–14^.

Pfs25 is one of the few leading targets for transmission-blocking vaccines, alongside Pfs48/45 and Pfs230^15^. Pfs25 is a cysteine rich protein consisting of four EGF-like domains. Pfs25 is expressed on the surface of developing ookinetes and is crucial for the development of oocysts within the mosquito midgut. The infectious sporozoite stage is produced in the oocysts which leads to the subsequent transmission of malaria in a new host. Several vaccine technologies have been evaluated to design Pfs25 vaccine candidates, including recombinant proteins, viral vectors, nanoparticles, and DNA plasmids^6,15–18^. However, the most advanced Pfs25 vaccine formulations have had limited success in clinical trials, eliciting weak immunogenicity and overall transmission reducing activity^16,17,19^.

Reasons for the poor overall efficacy of PfCSP and Pfs25 vaccine formulations in vaccine trials are not well understood. Some possibilities include suboptimal physicochemical nature of vaccine immunogens, poor representation of target epitopes in subunit vaccines, and sub-optimal overall immunogenicity using various approved adjuvants. These limitations emphasize the urgent need for alternate vaccine platforms for the rapid development and evaluation of vaccines addressing some of these limitations, and flexible enough to incorporate needed modifications to optimize for effective immunogenicity outcomes.

Technological advances continue to offer unique opportunities for the development of vaccines, such as the first approved use of mRNA-LNP vaccines against SARS-CoV2 with unprecedented success^20^. This success was propelled by the use of nucleoside-modified mRNA contributing to the enhanced protein production and induction of effective antibody responses *via* activation of germinal center responses (GC) and T follicular helper cells (Tfh)^21^. The development and use of lipid nanoparticle (LNPs) formulations for efficient delivery of mRNA to target cells further improved the overall effective immunogenicity^20,22^. mRNA vaccines have been shown to elicit robust immunogenicity at low doses for various diseases, including influenza, and SARS-CoV-2^20^, yet, the mRNA-LNP vaccine platform has not been fully evaluated for potential use in the development of malarial vaccine candidates^23,24^.

Previously, we showed that Pfs25 and PfCSP DNA vaccines administered with in vivo electroporation generated functionally effective immune responses in mice^25^. However, concerns about the likelihood of widespread implementation of vaccination using in vivo electroporation continue to drive the search for new technologies to improve nucleic acid-based vaccines. Therefore, we sought to evaluate the immunogenicity of two of the leading targets, Pfs25 and PfCSP, formulated as nucleoside-modified mRNA-LNP vaccines, and compare to Pfs25 and PfCSP DNA vaccines. We established the optimal immunogenic dose and immunization schedule for eliciting functionally effective immune responses, including protection against sporozoite challenge and *P. falciparum* transmission reduction to mosquitoes. An additional critical goal of our studies was to evaluate the possibility of co-immunization using multiple mRNA-LNP vaccines for immune targeting malaria parasites at various life cycle stages.

## Results

### Pfs25 mRNA-LNPs elicit high antibody titers in a dose-dependent fashion

**Fig. 1:**
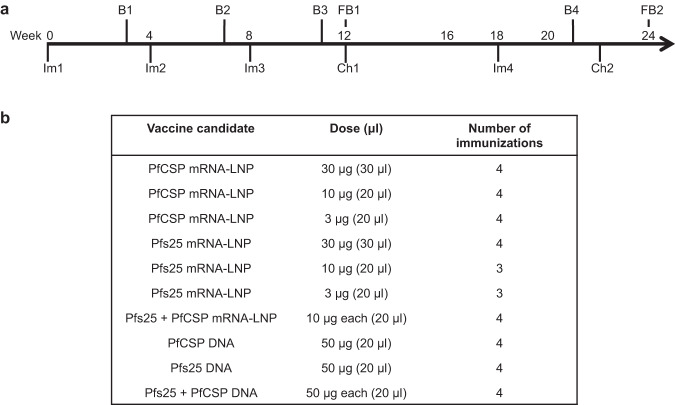
Timelines for mRNA immunization experiment. **a** Groups of female Balb/c mice were immunized (Im) with 3 μg, 10 μg, and 30 μg of mRNA-LNPs encoding either Pfs25 or PfCSP. Another group of mice received a combination of 10 μg of both Pfs25 and PfCSP mRNA-LNPs. In parallel, mice were also immunized with 50 μg of DNA plasmids encoding Pfs25 or PfCSP and a combination of 25 μg of both Pfs25 and PfCSP DNA plasmids. Immunizations were repeated as indicated, and test bleeds (B1-B4) and final bleeds (FB1 and FB2) were collected at indicated time points. Mice immunized with 3 μg and 10 μg Pfs25 mRNA-LNP were terminally bled at 12 weeks (FB1) while all other groups were terminally bled at 24 weeks (FB2). Mice immunized with PfCSP mRNA or DNA plasmids were challenged (Ch1 and Ch2) with sporozoites of PbPfCSP-GFPLuc to assess protection. 30 μg Pfs25 mRNA-LNP and Pfs25 DNA immunized mice were used as a negative control for sporozoite challenge experiments. **b** Table of immunization parameters corresponding to timeline in (**a**).

**Fig. 2:**
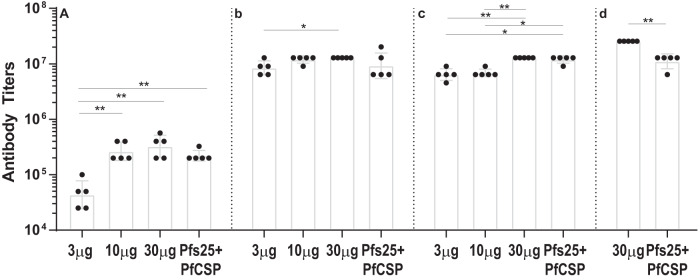
Pfs25-specific antibody titers determined by ELISA. mRNA-LNP immunization and blood collection schedules are shown in Fig. 1a. Sera (*N* = 5 per group) from bleeds at indicated time points [B1 in (**a**), B2 in (**b**), B3 in (**c**), and B4 in (**d**)] were analyzed using ELISA to determine antigen-specific antibody titers reported as the geometric mean endpoint titers with 95% confidence intervals. Endpoint titers were identified as the highest reciprocal serum dilution with an OD higher than a cutoff of the average OD plus three standard deviations of pre-immune sera replicates. Statistical analysis was performed using the Mann–Whitney U test (*p < 0.05; **p < 0.01).

To assess the antibody responses to Pfs25, female Balb/c mice were immunized with various doses (3, 10, and 30 μg) of Pfs25 mRNA-LNP and evaluated as shown in Fig. 1a. After the first immunization, the 3, 10, and 30 μg Pfs25 mRNA-LNPs elicited dose-dependent primary antibody responses with geometric mean titers of 43,528, 263,902, and 324,901, respectively (Fig. 2a). Administration of the second immunization of Pfs25 mRNA-LNP resulted in significant boosting of antibody responses with titers ~200-fold higher for the lowest dose (ELISA titer 8,444,851) and ~20-fold higher for the 10 μg (ELISA titer 11,942,822) and 30 μg (ELISA titer 12,800,000) Pfs25 mRNA-LNP doses (Fig. 2b). Additional immunization did not result in any further increase in antibody titers (Fig. 2c, d). While all doses elicited strong antibody production, the 30 μg Pfs25 mRNA-LNP elicited significantly higher titers compared with the 3 μg (*p* = 0.0079) and 10 μg (*p* = 0.0079) Pfs25 mRNA-LNP groups after three immunizations.

### PfCSP mRNA-LNPs elicit high antibody titers in a dose-dependent fashion

**Fig. 3:**
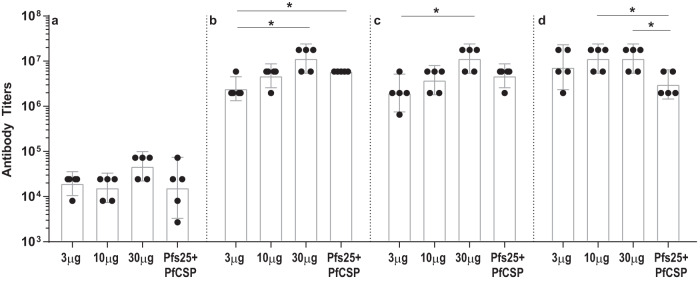
PfCSP-specific antibody titers determined by ELISA. mRNA-LNP immunization and blood collection schedules are shown in Fig. 1a. Sera from bleeds at indicated time points [B1 in (**a**), B2 in (**b**), B3 in (**c**), and B4 in (**d**)] were analyzed using ELISA to determine antigen-specific antibody titers reported as the geometric mean endpoint titers with 95% confidence intervals. Endpoint titers were identified as the highest reciprocal serum dilution with an OD higher than a cutoff of the average OD plus three standard deviations of pre-immune sera replicates. Statistical analysis was performed using the Mann–Whitney U test (*p < 0.05).

PfCSP mRNA-LNPs was evaluated at doses of 3, 10, and 30 μg, as shown in Fig. 1a. After one immunization, the 3, 10, and 30 μg PfCSP mRNA-LNP groups elicited PfCSP-specific antibody responses with geometric mean titers of 19,507, 15,658, and 46,976 (Fig. 3a). A second immunization resulted in 100 to 200-fold increases in antibody titers, with geometric means reaching 2,451,972, 4,740,109, and 11,415,247 for the 3 μg, 10 μg, and 30 μg PfCSP mRNA-LNP groups, respectively (Fig. 3b). Although there was a clear trend of a dose-dependent increase in the immunogenicity of PfCSP mRNA-LNP, the only statistically significant difference was between the 3 μg and 30 μg PfCSP mRNA-LNP groups (*p* = 0.0238). Any further immunization did not significantly alter antibody titers (Fig. 3c), with observed geometric mean titers of 1,968,300, 3,805,082, and 11,415,247 for the 3 μg, 10 μg, and 30 μg PfCSP mRNA groups, respectively.

As discussed below, we initially sought to evaluate protection against sporozoite challenge after three immunizations, however, the purified sporozoites used for challenge were found to be largely non-viable thus necessitating another challenge after a period of drug treatment and a fourth immunization (Fig. 1a). Geometric mean antibody titers after a fourth immunization in the 3 μg and 10 μg PfCSP mRNA-LNPs groups increased to 7,355,917 and 11,415,247, with the 30 μg mRNA-LNP group remaining at similar titers to those prior to the fourth immunization (Fig. 3d).

### Co-immunization with Pfs25 and PfCSP mRNA-LNP does not compromise antibody responses

A group of mice was immunized with a combination of both Pfs25 and PfCSP mRNA-LNPs to explore the immunogenicity of co-immunization of antigens targeting different parasite life cycle stages. Female Balb/c mice were co-immunized with 10 μg of Pfs25 and PfCSP mRNA-LNPs (Pfs25 + PfCSP mRNA-LNP), and the individual antigen-specific antibody titers were compared with antibody titers in mice immunized with Pfs25 mRNA-LNP or PfCSP mRNA-LNP alone. As shown in Figs. 2 and 3, Pfs25 and PfCSP-specific antibody titers after each immunization in the co-immunized group remained largely comparable to those obtained in mice immunized with either of the two immunogens individually. The only notable difference was higher Pfs25-specific titers after three immunizations in the combination group as compared to 10 μg Pfs25 mRNA-LNP (Fig. 2; *p* = 0.0079). Surprisingly, in the case of PfCSP-specific antibody responses, mice that had undergone experimental manipulations consisting of a challenge with largely non-viable sporozoites and drug treatment showed somewhat lower antibody titers after the fourth immunization in the combination group as compared to 10 μg PfCSP mRNA-LNP group (Fig. 3d). To rule out the possibility of any cross-reactivity between the two antigens, we tested sera from mice immunized with Pfs25 mRNA-LNPs for reactivity with PfCSP and vice versa using ELISAs. As shown (Supplementary Fig 3), immunized mice sera displayed antigen specific reactivity while the reactivity of Pfs25 immunized mice sera to PfCSP and the reactivity of PfCSP immunized mice sera to Pfs25 was comparable to that of pre-immune mouse sera used as a negative control for all ELISA.

### mRNA-LNPs elicit superior antibody responses than electroporation-mediated DNA plasmids

Previously we have shown that 50 μg of Pfs25 DNA administered with electroporation (EP) is immunogenic and efficacious in mice^26^. Another goal of our studies was to compare the relative immunogenicity differences between mRNA-LNP and DNA with in vivo EP for both Pfs25 and PfCSP. After each immunization, the antibody titers elicited by Pfs25 and PfCSP mRNA-LNPs, regardless of the vaccine dose, were superior to those elicited by immunization with Pfs25 and PfCSP DNA vaccines administered using in vivo EP (Supplementary Fig. 4a-b). Likewise, Pfs25 and PfCSP mRNA-LNPs combination (10 μg each) vaccine-elicited antibody responses were also superior as compared to antibodies elicited in mice co-immunized with a combination of Pfs25 and PfCSP DNA (25 μg each) administered with EP (Supplementary Fig. 4).

### Pfs25 mRNA-LNPs induce potent transmission-blocking antibodies

**Fig. 4:**
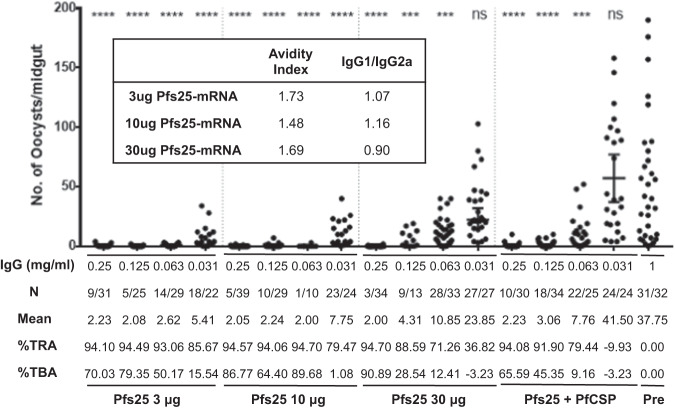
Evaluation of transmission reducing activity of antibodies induced by Pfs25 mRNA-LNPs. IgG was purified from pooled sera collected from mice immunized with 3 μg and 10 μg of Pfs25 mRNA-LNP (FB1) and 30 μg Pfs25 mRNA and 10 μg of both Pfs25+PfCSP mRNA-LNP combinations groups (FB2). Purified IgG were evaluated in mosquito standard membrane feeding assays (SMFA) at indicated final concentrations (0.25, 0.125, 0.63, and 0.031 mg/ml). Data points representing the oocyst counts from each mosquito, arithmetic means, and 95% confidence intervals are reported. N is the fraction of uninfected mosquitoes over the total number of mosquitoes dissected. %TRA is the mean reduction in oocysts in the presence of the test IgG compared with the IgG (1 mg/ml) from pre-immune mouse sera (Pre) used as a negative control. %TBA is the percent reduction in the proportion of infected mosquitoes in the presence of the test IgG compared with pre-immune IgG negative control (Pre). The inset shows IgG1/IgG2a isotype ratios and antibody avidity index for sera evaluated by SMFA. Statistical analysis was performed using the Mann–Whitney U test (ns *p* > 0.05; *p < 0.05; **p < 0.01; ***p < 0.001; ****p < 0.0001).

Due to the limited volume of sera collected, serum from final bleeds from each mouse was pooled to purify IgG for evaluation in SMFA. Sera (FB1 in Fig. 1a) from mice immunized three times with 3 μg and 10 μg Pfs25 mRNA-LNP were evaluated, while the 30 μg Pfs25 mRNA-LNP and (Pfs25 + PfCSP) mRNA-LNP combination groups were evaluated after four immunizations (FB2 in Fig. 1a). Initially, purified IgG from all the groups of the 3 μg, 10 μg, and 30 μg Pfs25 mRNA-LNP and Pfs25+PfCSP mRNA-LNP groups were evaluated at three concentrations (2, 1, 0.5 mg/ml) and all revealed greater than 94% TRA at all three concentrations of IgG tested. In subsequent experiments, lower concentrations of IgG (0.25, 0.125, 0.0625, 0.03125 mg/ml) were evaluated. IgG purified from pooled pre-immune mouse sera was used at 1 mg/ml as a negative control. IgG purified from the 3 μg and 10 μg Pfs25 mRNA-LNP immunized mice achieved greater than 90 % oocyst reduction (TRA) at IgG concentrations as low as 0.0625 mg/ml. Surprisingly, IgG from the 30 μg Pfs25 mRNA-LNP and Pfs25+PfCSP combination groups revealed relatively lower reduction with TRA ≥ 90% at 0.125 to 0.25 mg/ml IgG concentrations (Fig. 4).

To further characterize the anti-Pfs25 antibodies of sera that have transmission-blocking activity, Pfs25-specific antibody avidity and isotypes were evaluated using pooled serum of terminal bleeds of each group by ELISA (Fig. 4). The avidity indices of 1.73, 1.48, and 1.69 were similar among the pooled sera of the 3 μg, 10 μg, and 30 μg Pfs25 RNA-LNP immunized mice, respectively. We also compared Pfs25-specific IgG isotypes (expressed as IgG1/IgG2a ratios) in these sera and the results revealed balanced IgG isotypes with IgG1/IgG2a ratios of 1.20, 1.07, and 1.17 for the 3 μg, 10 μg, and 30 μg Pfs25 mRNA-LNP groups, respectively.

### Protection against sporozoite challenge after immunization with PfCSP mRNA-LNP

**Fig. 5:**
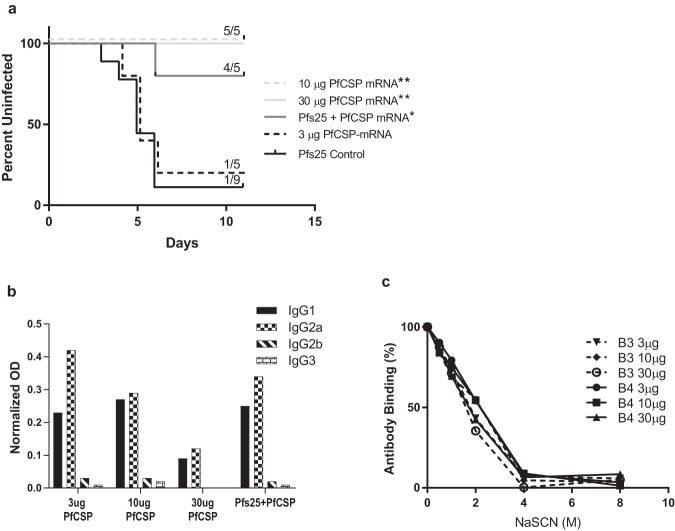
PfCSP mRNA-LNP induced responses in mice and protection against sporozoite challenge after four immunizations. Mice (*N* = 5 per group) immunized with 3 μg, 10 μg, 30 μg PfCSP mRNA-LNPs, or co-immunized with 10 μg of both Pfs25 and PfCSP mRNA-LNPs (as per Fig. 1a) were challenged with PbPfCSP-GFPLuc sporozoites and progression of infection was monitored by microscope examination of giemsa-stained blood smears for 11 days following challenge. Pfs25 immunized mice were used as negative controls. **a** Kaplan–Meier survival curves represent the percent of mice without detectable parasitemia following sporozoite challenge. Mice were considered completely protected if no parasitemia was detected by day 11. Statistical analysis was performed using log rank (Mantel-Cox) test (*p < 0.05; **p < 0.01). Antibodies from sera, collected one-week prior to the second challenge, bleed B4 in Fig. 1a, were characterized for antibody isotype (**b**) by ELISA using IgG1, IgG2a, IgG2b and IgG3 specific secondary antibodies. The optical densities (OD) of each isotype were normalized to the OD of total anti-mouse IgG. **c** Avidity was characterized by antibody-antigen dissociation curves resulting from treatment with different concentrations of NaSCN from sera collected from the Bleeds, B3 and B4, in Fig. 1a. The avidity index was calculated as the concentration (M) of NaSCN that resulted in 50% dissociation of bound antibodies.

The protection provided by the immunization with PfCSP was evaluated using an in vivo challenge model using sporozoites of transgenic *P. berghei* expressing PfCSP and luciferase (PbPfCSP-GFPLuc). For the first challenge (Ch1 in Fig. 1a), mice were inoculated with ~2000 PbPfCSP-GFPLuc sporozoites four weeks after the third immunization. Liver stage parasite burden was evaluated 42–44 h after the challenge by IVIS, quantifying the subsequent bioluminescence from the liver. None of the challenged mice, including negative controls, had any detectable liver stage burden suggesting that the sporozoites used for the challenge were not very viable or infectious. Additionally, only a fraction of Pfs25 immunized control mice (6/10) had any detectable blood-stage parasites by day 7 further corroborating that the viability of PbPfCSP-GFPLuc sporozoites was severely compromised during sporozoite isolation. To further evaluate the protective efficacy of the PfCSP mRNA LNP vaccine, mice were treated with chloroquine and sulfadiazine for six days to treat for any low-level parasitemia. Mice were given a fourth immunization after a rest period, and four weeks after the last dose of immunization mice were challenged again (Ch2 in Fig. 1a) and monitored for blood-stage parasitemia for 11 days. Five out of five mice of each 10 μg and 30 μg PfCSP mRNA-LNP group and four out of five mice of the Pfs25+PfCSP mRNA-LNP group were fully protected. In contrast, the 3 μg PfCSP mRNA-LNP group only had one out of five mice that achieved complete protection. Kaplan-Meier survival curves representing the percent of mice with detectable parasitemia each day following the challenge were used to assess any significant delays in blood-stage parasitemia. The survival curves of the 10 μg PfCSP mRNA-LNP (p = 0.004), 30 μg PfCSP mRNA-LNP (*p* = 0.004), and Pfs25+PfCSP mRNA- LNP groups (*p* = 0.0132), were significantly different to the Pfs25 mRNA-LNP immunized groups which served as a negative control for protection against infection by sporozoites. In contrast, the 3 μg PfCSP mRNA-LNP group showed no significant difference when compared to the Pfs25 mRNA-LNP control (*p* = 0.8319) (Fig. 5a).

To further characterize antibody responses at the time of protection, PfCSP-specific antibody avidity and isotypes were evaluated on pooled sera collected three weeks after the fourth dose (Fig. 5). The PfCSP-specific antibody avidity indices were similar between 3 μg, 10 μg, and 30 μg PfCSP mRNA-LNP groups, with avidity indices of 2.18, 2.20, and 1.72, respectively. To rule out influence of any potential impact of the transgenic parasites used in the first non-viable challenge, avidity of sera collected one week prior to the first challenge (B3 in Fig. 1a) was also assessed. The avidity indices for the 3 μg, 10 μg, and 30 μg PfCSP mRNA-LNP groups were 1.77, 2.01 and 1.59, respectively, which are within the experimental variability of the assay and similar to the avidity of sera collected three weeks after the fourth dose (B4 in Fig. 1a). In addition, the immune sera revealed IgG2a biased antibody isotypes with significantly pronounced IgG2a skewed isotypes in the sera from mice immunized with 3 μg and 30 μg PfCSP mRNA-LNP and the combination of Pfs25+PfCSP mRNA-LNP, with IgG1/IgG2a ratios of 0.75, 0.54, and 0.73, respectively.

### Evaluation of the minimum effective dose of Pfs25 mRNA-LNP and PfCSP mRNA-LNP

**Fig. 6:**
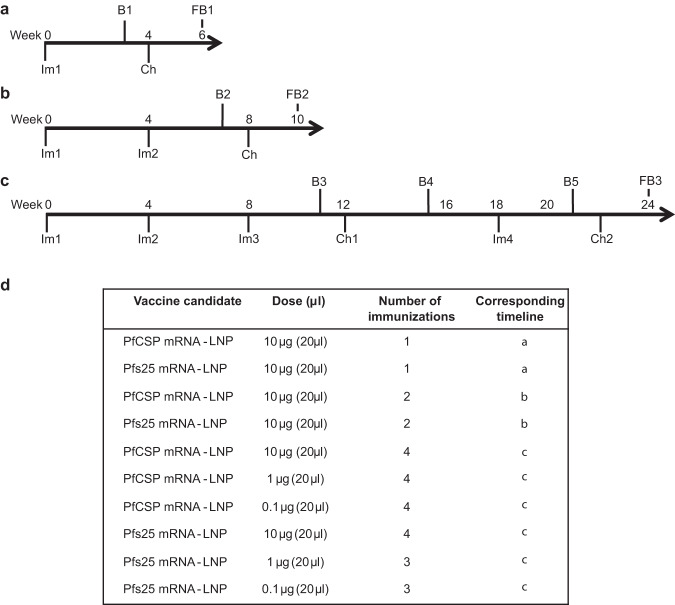
Experimental details for immunization experiment to evaluate lower vaccine doses and repeat immunizations. Groups of female Balb/c mice were immunized with 10 μg of either Pfs25 or PfCSP mRNA LNPs followed by a challenge with sporozoites after one (**a**), two (**b**), and three and four immunizations (**c**), as indicated. Two additional groups of mice for each mRNA-LNP were immunized with lower doses (0.1 μg and 1 μg) mRNA-LNPs to assess minimum immunogenic doses of Pfs25 or PfCSP mRNA-LNPs. B3 collected at 11 weeks from mice immunized with 0.1, 1.0 and 10.0 ug doses of Pfs25 mRNA-LNP were used in SMFA. Bleeds FB1 (**a**), FB2 (**b**) and B4 (**c**) were used in SMFA to evaluate transmission blocking activity in mice receiving one, two or three immunizations, respectively. **d** shows the immunization parameters corresponding to the timelines in (**a**–**c**).

**Fig. 7:**
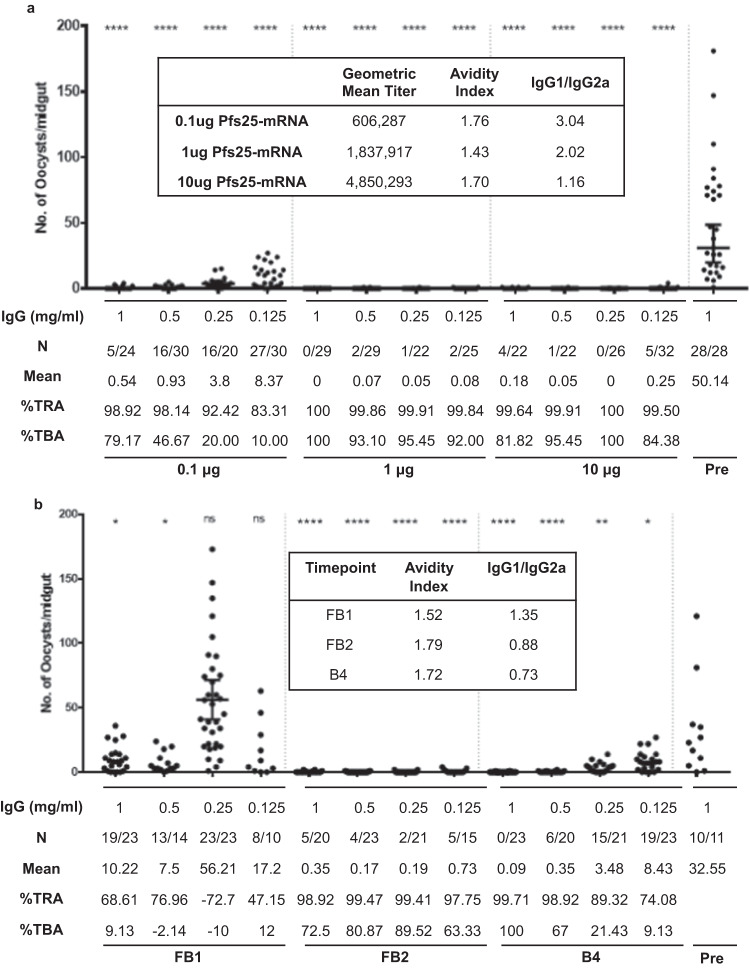
Transmission reducing activity of antibodies elicited by lower doses and varying immunization schedules of Pfs25 mRNA-LNP. **a** Purified IgG from pooled sera (bleed B3) from Balb/c mice immunized with 0.1 μg, 1 μg, and 10 μg Pfs25 mRNA-LNPs (Fig. 6c) were evaluated at indicated final concentrations by SMFA. The inset shows the geometric mean antibody titers, avidity indices, and IgG1/IgG2a ratios for the serum evaluated by SMFA. **b** SMFA data for purified IgG in FB1, FB2, and B4 (Fig. 6a–c) from Balb/c mice immunized with one, two and three doses of 10 μg of Pfs25 mRNA-LNP, respectively. The inset shows the antibody avidity indices and IgG1/IgG2a ratios for the serum evaluated by SMFA. Data points representing the oocyst counts from each mosquito, arithmetic means, and 95% confidence intervals are reported. N is the fraction of uninfected mosquitoes over the total number of mosquitoes dissected. %TRA is the mean reduction in oocysts in the presence of the test IgG compared with the IgG (1 mg/ml) from pre-immune mouse sera (Pre) used as a negative control. %TBA is the percent reduction in the proportion of infected mosquitoes in the presence of the test IgG compared with pre-immune mouse IgG negative control (Pre). Statistical analysis was performed using the Mann–Whitney U test (*p < 0.05; **p < 0.01; ***p < 0.001; ****p < 0.0001).

Due to the significant antibody responses elicited by the 3 μg mRNA-LNP group, we were interested in investigating whether lower doses of 1 μg or 0.1 μg mRNA-LNP would be immunogenic with effective antibody responses. Mice immunized three times (Fig. 6c) with 0.1 μg, 1 μg, and 10 μg Pfs25 mRNA-LNP revealed a dose-dependent antibody response with geometric mean titers of 606,287, 1,837,917, and 4,850,293, respectively (Fig. 7a inset). The 1 μg Pfs25 mRNA-LNP group had higher antibody titers than the 0.1 μg Pfs25 mRNA-LNP group, but the difference was not statistically significant. However, the antibody titers in the mice immunized with 10 μg Pfs25 mRNA-LNP were significantly higher than the 1 μg (*p* = 0.0476) and 0.1 μg (*p* = 0.0079) Pfs25 mRNA-LNP groups.

SMFA was conducted to evaluate the functional efficacy of the antibodies elicited by lower doses of Pfs25 mRNA-LNP (Fig. 7a). Purified IgG was evaluated at 1.0, 0.5, 0.25, and 0.125 mg/ml final concentrations, with IgG purified from pre-immune sera used as a negative control at 1 mg/ml. The IgG purified from the 1 μg and 10 μg Pfs25 mRNA-LNP immunization groups elicited nearly 100% blocking at the lowest concentration of 0.125 mg/ml IgG tested. IgG from the 0.1 μg Pfs25 mRNA-LNP immunized mice, appeared to be relatively less potent, eliciting 98–99% TRA only at higher (0.5 to 1.0 mg/ml) IgG concentrations, with decreasing TRA at lower concentrations (<0.25 mg/ml) of IgG tested.

We also characterized the antibody isotype and avidity in the sera used for the SMFA (Fig. 7a, inset). The avidity indices for the 0.1 μg, 1 μg, and 10 μg groups were not different from those seen earlier when higher immunizing doses up to 30 μg were evaluated (Fig. 4). Strikingly, the antibody isotype analysis did reveal rather unexpected IgG1 skewed responses with IgG1/IgG2a of 3.04, and 2.02 in the mice immunized with 0.1 μg and 1.0 μg Pfs25 mRNA-LNP, respectively. Consistent with the previous analysis, the 10 μg Pfs25 mRNA-LNP group elicited a balanced response, with an IgG1/IgG2a ratio of 1.16.

**Fig. 8:**
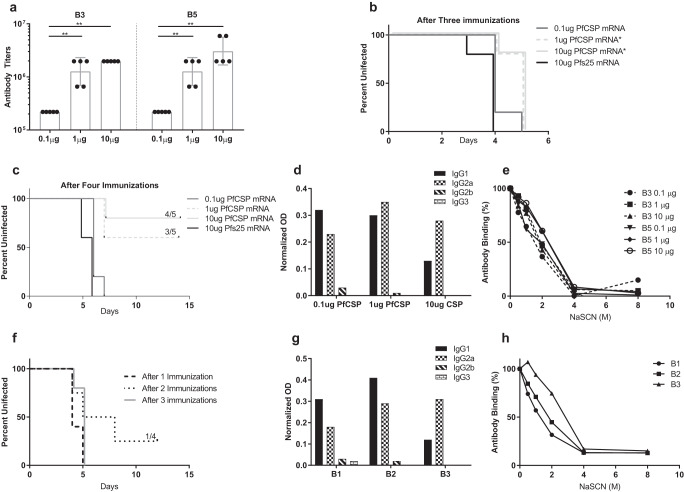
Evaluation of varying vaccine doses and immunization schedules of PfCSP mRNA-LNPs. Details of immunization and challenge are described schematically in Fig. 6a–c. **a** Anti-PfCSP antibody responses from B3 and B5 (Fig. 6c) were analyzed by ELISA and are reported as the geometric mean endpoint titers with 95% confidence intervals. **b** Kaplan-Meier survival curves representing outcome of sporozoite challenge after three immunizations with PfCSP mRNA-LNP (*N* = 5 per groups). Mice after challenge were observed for 12 days by microscopic examination of giemsa-stained blood smears. Following the first challenge, mice were treated and immunized a fourth time and challenged as shown in Fig. 6c. **c** Kaplan-Meier survival curves after repeat challenge (*N* = 5 per group). Panels **d** shows antibody isotypes from the bleed, B5, and (**e**) shows antibody-antigen avidity index from the bleeds, B3 and B5 (Fig. 6c). Panels **f, g**, and **h** show Kaplan-Meier survival curves for mice immunized with 10 μg PfCSP mRNA-LNP (*N* = 5 per group except mice receiving 2 immunizations, *N* = 4) and challenged after each immunization (Fig. 6a–c), anti-PfCSP antibody isotypes and anti-PfCSP avidity index, respectively. Mann–Whitney U test was used for statistical analysis of antibody titers, while log rank (Mantel Cox) test was used for the analysis of survival curves (*p < 0.05; **p < 0.01).

As above, we also re-examined the immunogenicity of the lower doses of the PfCSP mRNA-LNP vaccine. Mice were immunized with 0.1 μg, 1 μg, or 10 μg PfCSP mRNA-LNP (schedule in Fig. 6c), followed by an evaluation of antibody responses in bleeds (B3 and B5) and protective efficacy. There was a sharp increase in anti-PfCSP antibody response in bleed B3 from 0.1 μg to 1.0 μg, with geometric mean titers of 218,000, 1, 268,600, and 1,968,300 in mice immunized with three doses of PfCSP mRNA-LNP, respectively. Immunization with another dose did not appreciably boost antibody responses in the bleed B5 in the various groups, except for the 10 μg PfCSP mRNA-LNP group, which showed a ~50% increase in antibody titers (Fig. 8a).

To assess the protective efficacy of immunization with low doses of PfCSP mRNA-LNP, mice were challenged with sporozoites of PbPfCSP-GFPLuc. Even though significant levels of antibodies were detected, mice in the various dose groups immunized three times were not protected against the challenge infection. Blood-stage parasitemia was detected beginning on day 3 post-challenge in all the mice, however, mice immunized with 1 μg and 10 μg PfCSP mRNA-LNP revealed a one-day difference in the detection of parasites when compared with the Pfs25 mRNA-LNP control group, resulting in significant differences in survival curves between the Pfs25 control group and the 1 μg (*p* = 0.0145) and 10 μg (*p* = 0.0145) PfCSP mRNA-LNP groups (Fig. 8b). Given these results, we drug cured the challenged mice and immunized one more time, and subsequently challenged as per the schedule in Fig. 6c. As shown in Fig. 8c, 4 out of 5 mice in the 10 μg PfCSP mRNA-LNP group and 3 out of 5 mice in the 1 μg PfCSP mRNA-LNP group showed complete protection over 14 days period of monitoring blood-stage parasitemia. However, mice immunized with the 0.1 μg PfCSP mRNA-LNP were not protected against the challenge infection. The survival curves of uninfected mice of the 1 μg PfCSP mRNA-LNP group (*p* = 0.0031) and the 10 μg PfCSP mRNA-LNP group (*p* = 0.0031) were statistically different compared to the Pfs25 mRNA-LNP control group (Fig. 8c). There was no evidence that the survival curve of the 0.1 μg group was different from the Pfs25 mRNA-LNP negative control group (*p* = 0.0951). Results in Fig. 8d present evidence for a shift in antibody isotype profile from a balanced isotype response in the mice immunized with 0.1 and 1.0 μg dose which becomes IgG2a skewed in mice immunized with the higher 10 μg dose. Apart from this isotype profile shift, no significant difference was observed in the avidity index among the 0.1 μg, 1 μg and 10 μg PfCSP mRNA-LNP groups with indices of 1.72, 2.33 and 2.40. The avidity indices of sera collected prior to any challenge were 1.52, 1.91 and 1.80 (Fig. 8e) and the difference between bleeds are within the experimental variability of the assay, thus ruling out any modulation as a result of the exposure to the transgenic parasites used in the first challenge.

During the course of these studies, we also evaluated immunogenicity parameters and functional protective efficacy in mice immunized with different immunization regimens (1, 2, and 3 immunizations) of 10 μg of Pfs25 or PfCSP mRNA-LNPs (Fig. 6a–c). Figure 7b shows the results of transmission-blocking SMFA in the presence of purified IgG from mice immunized with 10 μg Pfs25 mRNA-LNP. IgG from sera after a single immunization (FB1) revealed only modest TRA. However, IgG from mice immunized two (FB2) or three times (B4) exhibited robust TRA. The Fig. 7b inset also shows data on antibody avidity and isotype for each bleed with results similar to those in earlier studies (Fig. 4). Results of protection against sporozoite challenge in groups of mice immunized with various regimens of 10 μg PfCSP mRNA-LNP are shown in Fig. 8f. Consistent with previous results showing the importance of a four-dose regimen (Figs. 5 and 8c), one, two, or three immunizations were not sufficient in giving any significant protection against sporozoite challenge (Fig. 8f). Further analysis of immune sera did reveal an antigen-specific effect of improved avidity with subsequent immunization. We first tested pooled sera after each immunization and then reconfirmed by testing individual mouse serum. As shown in Fig. 8h, the mean avidity indices increased from 0.908 to 1.25, and 1.886 in mice receiving one, two, or three immunizations of 10 μg PfCSP mRNA-LNP, respectively. A significant difference was detected between the mice that received one immunization and mice that received three immunizations (*p* = 0.0090). The isotype analysis revealed once again IgG2a skewed antibodies after three immunizations (Fig. 8g).

### Analysis of splenic T cell and B cell responses in mice immunized with Pfs25 and PfCSP mRNA-LNPs

**Fig. 9:**
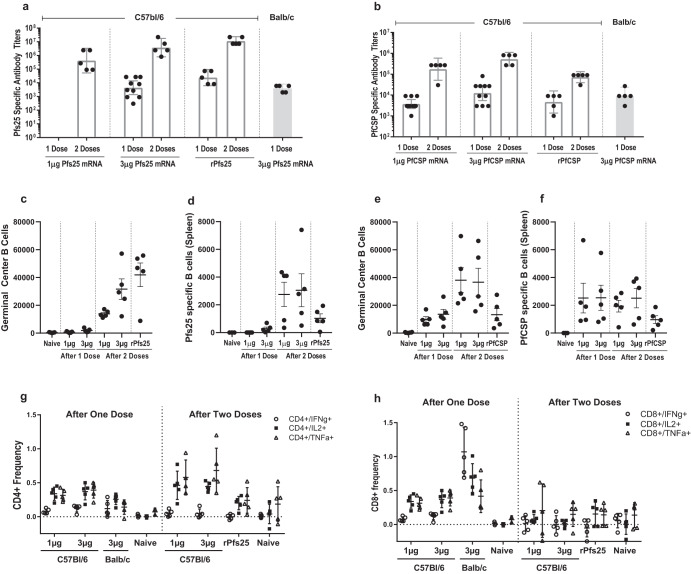
Evaluation of cellular responses in mice immunized with Pfs25 and PfCSP mRNA-LNPs. Groups of C57Bl/6 and Balb/c mice (*N* = 5) were immunized with 1 μg and 3 μg of either Pfs25 or PfCSP mRNA-LNPs following one or two immunizations. Additional groups were immunized with two doses of 10 μg of rPfs25 and rPfCSP formulated with Alhydrogel, as positive controls. **a** Anti-Pfs25 antibody responses, and **b** Anti-PfCSP antibody responses evaluated by ELISA are reported as the geometric mean endpoint titers with 95% confidence intervals. Germinal center B (GCB) cells and antigen specific B cells were quantified by flow cytometry. The reagents used are found in Supplementary Table 1. and the gating strategy is reported in Supplementary Fig. 1. Panels **c** and **d** show quantification of GCB cells and Pfs25-specific B cells respectively in Pfs25 mRNA-LNP immunized mice. Panels **e** and **f** show the quantification of GCB cells and PfCSP-specific B cells, respectively in PfCSP immunized mice. Additionally for the Pfs25 immunized mice, the harvested splenocytes were stimulated with a pool of overlapping peptides of Pfs25 for 6 h and assessed by flow cytometry for T cell responses. The frequency of stimulated cytokine-producing T cell subpopulations is reported. Panel **g** shows CD4 T cell responses and panel **h** shows the CD8 T cell responses. Error bars for panels (**c-h**) represent the standard error of the mean.

The cellular responses of each mRNA-LNP were investigated by the immunization of female Balb/c and C57Bl/6 mice. The C57Bl/6 mice were immunized with either one or two doses of either Pfs25 or PfCSP mRNA-LNP (1 μg or 3 μg) while the Balb/c mice were immunized with a single 3 μg dose of Pfs25 or PfCSP mRNA-LNP. Additionally, mice immunized with either recombinant Pfs25 or PfCSP proteins administered with the adjuvant, Alhydrogel, were used as a positive control. To confirm the immunogenicity of the immunization, antigen-specific antibody responses were determined from sera collected two weeks following each immunization (Fig. 9a and b). The C57Bl/6 mice immunized with 1 μg of Pfs25 mRNA-LNP were unresponsive two weeks following the first immunization while the C57Bl/6 mice immunized with 3 μg Pfs25 mRNA-LNP elicited antibody titers comparable to antibody responses of Balb/c mice. After a second immunization, antibody titers for all groups increased as expected.

The GC B cells and antigen-specific B cells were measured via flow cytometry of splenocytes collected from the immunized C57Bl/6 mice. Spleens collected from unimmunized mice were used as a control. The number of GC B cells was very low following one immunization and increased significantly following the second dose. In comparison, the amount of GC B cells of mRNA-LNP immunized mice were lower than GC B cells of rPfs25 immunized mice (Fig. 9c). The same trend occurred with Pfs25-specific B cells, with very low number of cells observed after the first immunization and higher amounts observed following the second immunization (Fig. 9d). For PfCSP immunized mice, modest amount of GC B cells was observed in mice immunized with one dose of PfCSP mRNA-LNP and increased significantly following the second dose (Fig. 9e). Two doses of PfCSP mRNA-LNPs elicited higher GC B cells than two doses of rPfCSP with Alhydrogel. Interestingly PfCSP-specific B cells were very low for all groups, yet were similar between all PfCSP mRNA-LNP immunized mice and did not increase following a booster dose (Fig. 9f). Additionally, all PfCSP mRNA-LNP immunized mice had higher PfCSP-specific B cells than those of rPfCSP immunized mice.

For Pfs25 mRNA-LNPs, T cell responses were evaluated. Splenocytes were harvested and cultured with or without stimulation with Pfs25 overlapping peptide pools and the frequency of proliferated splenic T cells producing key cytokines as a result of stimulation was determined. The C57Bl/6 mice immunized with one dose of Pfs25 mRNA-LNP elicited extremely low IL4 and IL5 producing CD4 T cells, while IFNγ, IL2, and TNFα producing CD4 T cells were elevated, showing a Th1 skewed response. C57Bl/6 mice immunized with two doses of Pfs25 mRNA-LNP elicited elevated IL2 and TNFα producing CD4 T cells compared with mice those receiving one dose. In contrast IFNγ producing CD4 T cells were similar between the one dose and two dose regimens (Fig. 9g). In C57Bl/6 mice, the CD8 T cells producing IFNγ, IL2, and TNFα were elevated in mice that received one dose but were low in mice that received two doses (Fig. 9h).

Additionally, strain-specific T cells responses were observed. The Balb/c mice which received one 3 μg dose had IFNγ producing CD4 T cells that were similar to the C57Bl/6 mice which received one 3 μg dose while IL2 and TNFα producing CD4 T cells were slightly lower (Fig. 9g). In contrast, IFNγ and IL2 producing CD8 T cells were significantly higher in Balb/c mice compared with C57Bl/6 mice (Fig. 9h). Thus a clear difference in T cell responses was observed between the two mouse strains.

## Discussion

Malaria vaccine development has focused on antigens expressed during various stages of the life cycle of the parasite. Malaria transmission depends upon the development of intraerythrocytic sexual stages, ingestion by female anopheline mosquitoes, and subsequent sexual development in mosquitoes leading to the formation of sporozoites. An infected *Anopheles* mosquito initiates the malaria infection cycle by injecting sporozoites into the host which invade hepatocytes leading to pathogenic blood-stage infection. Hence immune interventions aimed at blocking the development of both the liver stage and sexual stage are expected to provide a more effective strategy to protect against malaria infection and transmission. A transmission-blocking vaccine (TBV) approach targeting antigens in the sexual stages (i.e. male and female gametocytes and gametes) and the mosquito stages of the parasite (i.e. zygote and ookinete) is believed to be of central importance in malaria elimination efforts. The primary goal of the studies reported here was to evaluate the mRNA-LNP platform for the development of vaccines targeting multiple vulnerable life cycle stages of the malaria parasite. The studies focused on immunogenicity and functional (protective) activity of immune responses elicited by mRNA-LNP encoding the two most prominent *P. falciparum* target antigens; PfCSP (circumsporozoite protein present on the surface of sporozoites initiating infection) and Pfs25 (a target antigen for the development of a TBV).

We show that Pfs25 mRNA-LNPs are highly immunogenic at the various doses evaluated. Doses ranging from 0.1 μg to 30 μg elicited strong antibody responses when given in a 3-dose immunization regimen, spaced 4 weeks apart. Antibody responses tend to be modest following the priming dose which increased significantly following a booster dose. The ability to effectively prime immune responses is also supported by the quantification of germinal center B cells, where we saw a low level of germinal center B cells in the spleen following a single dose which increased significantly after a booster dose. Additionally, we observed robust CD4 and CD8 T cell responses in both C57Bl/6 and Balb/c mice. Additionally, strain-specific responses were observed, where Balb/c mice had a significantly stronger CD8 T cell response compared with C57Bl/6 mice. It is currently understood that antibodies to Pfs25 mediate the transmission-blocking response^15,27^, therefore it is unknown how elevated CD8 T cells may impact TRA and TBA and warrant further studies.

For all doses of Pfs25 mRNA-LNP given in a three-immunization regimen, the SMFAs revealed potent TRA and TBA in the presence of low IgG concentrations. Even vaccine doses as low as 0.1 μg Pfs25 mRNA-LNPs elicited significant TRA, which improved further as higher vaccine doses were tested. A low dose of 1 μg appeared to optimally provide both high immunogenicity and potent functional mosquito transmission-blocking activity. Immunization with 1 μg and higher amounts of Pfs25 mRNA-LNP revealed highly significant transmission-blocking activity with greater than 90% transmission-blocking activity by IgG concentration of 0.125 mg/ml. Additionally, after three immunizations, the 10 μg Pfs25 mRNA-LNPs elicited ~2.5 greater antibody titers compared with the 1 μg Pfs25 mRNA-LNPs, however, both their respective TRA and TBA functional activities were comparable. Further studies looking at optimization of immunogenicity revealed that even though antibodies elicited by a single dose of a higher amount (10 μg) of mRNA-LNPs elicited transmission reducing antibodies, a booster immunization was invariably required for potent functional activity. It is known that modified mRNA-LNP vaccines induce potent Tfh cell responses that drive potent GC responses. Modified mRNA-LNP induced Tfh cells also drive affinity maturation and responses to subdominant epitopes^21,28^. We hypothesize that similar protective responses are induced by Pfs25 mRNA-LNPs, which will be investigated in future studies.

Interestingly, mice immunized with the highest dose (30 μg) revealed superior antibody titers as compared to the 3 μg and 10 μg Pfs25 mRNA-LNP groups however the antibodies appeared to be less effective in TRA. Because these mice had received a fourth immunization and were used as a control group for parasite challenge (studies discussed below) we do not know if the active infection resulted in the loss of potency of antibodies in MFA. We tried to seek an understanding of the impact of parasite challenge through the analysis of antibody avidity and antibody isotypes (IgG1/IgG2a ratio) and found that they were not significantly modulated by parasite challenge. We hypothesize that the parasite challenge is modulating the humoral response by eliciting antibodies of other specificities which may lead to a reduction in the relative proportions of Pfs25-specific antibodies in the purified IgG, reflected in reduced TRA activity in the SMFA. We do not know if repeat immunizations using higher doses are leading to overall reduced functional effectiveness.

As with Pfs25 mRNA-LNP, PfCSP mRNA-LNP elicited moderate antibody responses following one immunization, and robust responses after each subsequent booster dose. Although antibody titers were high, immunized mice were not completely protected against sporozoite challenge following three immunizations. We did notice a significant 1-day delay in the blood-stage parasitemia in the 1 μg and 10 μg PfCSP mRNA-LNP vaccine groups compared with the negative control group, suggesting that some level of protection was conferred. However, due to the lack of data on liver-stage parasite burden, the level of partial protection could not be quantified. A one day delay however corresponds to a ~ 90% reduction in liver stage burden. Encouraged by these observations, mice immunized with PfCSP mRNA-LNPs were given another booster immunization and sporozoite challenge to observe whether another immunization would improve protection. Interestingly, mice immunized with four doses revealed a significant percentage of mice eliciting complete protection. Our findings were similar to previous studies, where PfCSP mRNA formulated with different LNPs was found to be highly immunogenic and provide partial protection in mice^23^. Additionally, Our studies suggest that PfCSP mRNA-LNP immunogenicity requires greater than three immunizations, as has been observed in humans with RTS,S/AS01^29^. However, because these mice had been challenged once prior to the fourth immunization, we cannot rule out that there was some contribution of heterologous boosting as transgenic *P. berghei* sporozoites used for the challenge express PfCSP protein which might provide an unintended heterologous protein boost, and may provide a rationale for a heterologous boosting strategy used for PfCSP vaccine evaluation^30–32^.

Apart from exploring the immunogenicity of the mRNA-LNP vaccines encoding Pfs25 and PfCSP alone, another key goal of our studies was to evaluate the possibility of co-immunization of mRNA-LNPs affecting different parasite life cycle stages to effectively perturb propagation of infection and transmission cycles. The co-immunization of Pfs25 mRNA-LNP and PfCSP mRNA-LNPs elicited comparable antigen-specific antibody responses to the single antigen mRNA-LNPs. Both the antibody responses and the functional activities after immunization with single antigen or combination vaccines were found to be comparable thus providing supporting evidence in favor of the possibility of combining multiple vaccines without any negative consequences using the mRNA-LNP platform. Furthermore, although PfCSP mRNA-LNP was not completely protective, the overall efficacy when in combination with Pfs25 mRNA-LNP may be enhanced over multiple generations, as was shown in a multigenerational *P. berghei* mouse model using a combination of passively transferred partially effective antibodies against P25 and PCSP^33^.

Overall, we show that the use of the mRNA-LNP platform for targeting the malarial antigens, Pfs25 and PfCSP, is highly effective in eliciting protective immunogenicity outcomes. The Pfs25 mRNA-LNP is extremely promising as both low dose immunizations and short prime-boost regimens elicited extremely potent functional activity. The PfCSP mRNA-LNP on the other hand seemed to require multiple booster immunization or perhaps a modified strategy that incorporated mRNA prime and a heterologous protein boost to elicit complete protection in mice. While a vaccine targeting sporozoite will prevent or reduce the development of blood-stage parasites including gametocytes in an infected person, a TBV will block the sexual reproduction of the gametocytes in the mosquito. A combination of vaccines targeting both the infection stage and sexual/midgut stages is expected to provide effective ways to interrupt malaria transmission, which is critical for achieving elimination goals.

## Methods

### Study design

For the initial evaluation of the mRNA-LNPs, groups (*n* = 5) of 6–8 week-old female Balb/c mice (Charles River) were immunized with various doses (3, 10, 30 μg) of either PfCSP mRNA-LNP or Pfs25 mRNA-LNP alone or were co-immunized with 10 μg of both mRNA-LNPs. In parallel, groups of Balb/c mice were also immunized with 50 μg of DNA encoding PfCSP or Pfs25 alone or a combination of both PfCSP and Pfs25. DNA plasmids were administered using in vivo electroporation with a BTX Agile Pulse electroporator. Mice were immunized with 3 or 4 doses given at 4-week intervals or as described in Fig. 1. Mice immunization schedules and timings of collection of blood and parasite challenges in various experiments are shown schematically in Fig. 1. In the follow-up experiment, lower doses (0.1, 1, and 10 μg) and different immunization regimens (1, 2, and 3 immunizations using a fixed dose of 10 μg) of mRNA-LNPs were evaluated in groups of 6–8 week old female Balb/c mice using immunization schedules as indicated (Fig. 6a–c). For evaluation of cellular responses, studies included groups (*n* = 5) of C57Bl/6 and Balb/c mice. Mice were immunized with Pfs25 mRNA-LNP, PfCSP mRNA-LNP, or, rPfs25 and rPfCSP proteins formulated with the adjuvant, Alhydrogel (2% aluminum hydroxide gel suspension), and splenocytes were harvested for evaluation following either the priming dose or the subsequent booster dose administered after 15 days.

For all immunizations, mice were anesthetized with isoflurane and were immunized *via* intramuscular injection in the left anterior tibialis. Typically, blood was collected three weeks following each immunization from the tail vein and at the end of each study by either cardiac puncture or retro-orbital bleed for isolation of serum for various tests (Figs. 1 and 6). In vivo experiments in mice were approved by IACUCs of the George Washington University and University of Pennsylvania. All experimentation adhered to the Guide for the Care and Use of Laboratory Animals by the National Research Council. Mice were housed and cared for in Association for Assessment and Accreditation of Laboratory Animal Care International (AAALAC)-accredited facilities.

### Production of mRNA-LNP vaccines

Codon-optimized coding sequences of Pfs25 (AF193769.1) and PfCSP (XP_001351122.1) were synthesized and cloned into an mRNA production plasmid as described^34^. Modified nucleoside containing mRNAs were produced to contain 101 nucleotide-long poly(A) tails and using m1ψ-5’-triphosphate instead of UTP. In vitro transcribed mRNAs were capped co-transcriptionally using the trinucleotide cap1 analog, CleanCap. mRNA purified by cellulose purification^35^ were analyzed by agarose gel electrophoresis and were stored frozen at –20 °C until being encapsulated in LNPs using a self-assembly process. Briefly, an ethanolic lipid mixture of ionizable cationic lipid, phosphatidylcholine, cholesterol, and polyethylene glycol-lipid was rapidly mixed with an aqueous solution containing mRNA at acidic pH^36^. The LNP formulation used in this study is proprietary to Acuitas Therapeutics (US patent US10,221,127). The RNA-loaded particles were characterized and subsequently stored at –80 °C at an RNA concentration of 1 mg/ml and a total lipid concentration of 30 mg/ml. The mean hydrodynamic diameter of mRNA-LNPs, measured by dynamic light scattering using a Zetasizer Nano ZS (Malvern) was ~80 nm with a polydispersity index of 0.02-0.06 and encapsulation efficiency of 95%.

### Production of DNA vaccines

Pfs25 DNA plasmid was constructed by cloning a codon-optimized (GenScript) Pfs25 sequence lacking the N-terminal signal sequence, C-terminal anchor sequence, and mutated N-linked glycosylation sites into the DNA vector VR1020^26^. PfCSP DNA plasmid was constructed similarly by cloning a codon-optimized full-length PfCSP sequence (Genscript) into the vector, VR1020. The two plasmids were resuspended in endotoxin-free water at 2.5 mg/ml concentrations. For the co-immunization of Pfs25 and PfCSP DNA vaccines, each plasmid was concentrated, mixed in equal amounts, and diluted to a final concentration of 5 mg/ml.

### Parasite challenge

Transgenic *P. berghei* (ANKA) parasites expressing *P. falciparum* CSP, GFP, and luciferase (PbPfCSP-GFPLuc) were used for the challenges^37^. Infectious sporozoites were produced by feeding *Anopheles stephensi* mosquitoes on an infected Swiss Webster (CFW) mouse. 20-23 days post-infection, mosquito salivary glands were dissected, placed in a 1.5 ml microcentrifuge tube containing HBSS with 2% FBS, and teased using a pestle to release sporozoites. The sporozoite suspension was filtered using a 100 μm mesh to remove debris. Mice were challenged with ~2000 sporozoites intravenously into the tail vein. For the first challenge only, parasite liver burden was assessed by IVIS, 42–44 h post-challenge. 100 μl RediJect D-luciferin was injected intraperitoneally into mice, and the resulting bioluminescence intensity of each mouse liver was quantified using an IVIS Lumina III in vivo imaging system. Following every challenge, blood-stage parasitemia was monitored by microscopic evaluation of gimesa-stained blood smears starting three days post-challenge. Blood-stage parasitemia was monitored for at least 11 days or until 5-10% parasitemia is reached. Complete protection was defined when no parasitemia was detected for up to 11 days following the challenge. In some experiments, infected mice were administered a combination of oral sulfadiazine (30 mg/l) in drinking water and daily intraperitoneal injections of chloroquine (20 mg/kg body weight) for six days to cure mice. The clearance of parasites was confirmed by microscopic examination of gimesa-stained blood smears.

### IgG purification

Total IgG from sera was purified using Protein-G Sepharose beads (Invitrogen). Pooled sera were incubated with beads in an equal volume of binding buffer (1.5 M glycine, 3 M NaCl, pH 9) for 3-4 h at 4 °C. Beads were washed with binding buffer and the bound IgG was eluted with 0.2 M glycine pH 2.5 and collected into tubes containing 1 M Tris (pH 9) to immediately neutralize the pH. Purified IgG was concentrated to 4-8 mg/ml and stored at −20 °C.

### *P. falciparum* Gametocyte culture

Mature gametocytes of the human malaria parasite, *P. falciparum* (NF54), were cultured as described earlier^38,39^. Parasites were cultured using O + human erythrocytes at 4% hematocrit in parasite culture medium (RPMI 1640 supplemented with 25 mM HEPES, 10 mM glutamine, 0.074 mM hypoxanthine, and 10% O + human serum). Gametocyte cultures were initiated at 0.5% parasitemia from low-passage stock and were maintained up to day 18 with daily medium changes. Culture plates were incubated at 37 °C in a microaerophilic environment inside a candle jar. The use of human erythrocytes to support the growth of *P. falciparum* was approved by the Internal Review Board (IRB) of the Johns Hopkins University Bloomberg School of Public Health (#NA 00019050).

### Standard membrane feeding assay (SMFA)

The functional activity of transmission-blocking vaccines was evaluated by SMFA^26^. Purified IgG was diluted to desirable test concentrations in a mixture of approximately 0.3% *P. falciparum* (NF54) gametocytes, human red blood cells (50% hematocrit), and normal human sera. The antibody-parasite mixture was fed to 25-40, 4–6-day old *An. stephensi* mosquitoes (starved 6–8 h) using glass membrane feeders maintained at 37 °C for 15 min. Unfed mosquitoes were removed and blood-fed mosquitoes were maintained at 27–28 °C with 70–80% relative humidity for 7–8 days. Mosquito midguts were dissected, and oocysts were enumerated after staining with 0.5% mercurochrome for 15–20 min. Transmission reducing activity is defined as the percent reduction in mean oocysts between the test IgG-fed mosquitoes and the control IgG-fed mosquitoes. Transmission blocking activity is defined as the percent reduction in the proportion of infected mosquitoes between the test IgG fed and control IgG fed groups.

### Recombinant Pfs25 and PfCSP and Synthetic Peptides

Recombinant Pfs25 protein was purified as reported previously^40^. rPfs25 was expressed in BL21 (DE3) E. coli. The bacterial pellet was lysed by microfluidization, washed in PBS pH 7.4 and resuspended in 100 mM Tris pH 12 while rocking for 20 min, then centrifuged (Beckman Avanti J-E, JA-25.50) at 20,442 × *g* for 30 min at 4 °C. The supernatant was diluted 10 fold with 100 mM Tris pH 8 and the pH was adjusted to pH 6.8. The supernatant was incubated with Ni-NTA beads (Qiagen) overnight at 4 °C. The beads were washed with, 20 mM imidazole in 100 mM Tris pH 6.9, 30 mM imidazole in 100 mM Tris pH 6.9, 10% glycerol in 100 mM Tris pH 6.9, and a 10:1 ratio of reduced/oxidized glutathione in 100 mM Tris pH 6.9 with 10% glycerol. rPfs25 was eluted with 100 mM imidazole. rPfs25 was incubated for 30 min with 10:1 ratio of glutathione and dialyzed in PBS pH at 4 °C. Recombinant PfCSP was produced by expression in E. coli. Codon harmonized PfCSP sequence (lacking N-terminal signal and C-terminal GPI anchor sequence) with a 6x histidine tag fused at the 5’ end with a spacer (PGGSGSGT) was synthesized (GenScript) and cloned into a pET (K-) expression vector and transformed into BL21 (DE3) E. coli. Protein expression was induced with 0.1 mM IPTG. Bacteria were harvested by centrifugation (Beckman Avanti J-E, JLA9.1) at 7460 × *g* for 30 min at 4 °C. The cell pellet was resuspended in lysis buffer (50 mM NaH_2_PO_4_, 300 mM NaCl, 20 mM imidazole pH 8) and lysed via microfluidization. Lysed bacteria were centrifuged (Beckman Avanti J-E, JA-25.50) at 20,442 × *g* for 30 min at 4 °C, and the supernatant was clarified by filtration (0.45 um) and incubated with Ni-NTA beads (Qiagen) at 4 °C, overnight. Ni-NTA beads were washed with the wash buffer (50 mM NaH_2_PO_4_, 300 mM NaCl, 20 mM imidazole pH 8) containing 0.25% Sarkosyl followed by the wash buffer alone. Bound protein was eluted from the beads using 250 mM imidazole in phosphate buffer (50 mM NaH_2_PO_4_, 300 mM NaCl, pH 8). Buffer exchange with PBS pH 7.4 was conducted using Amicon Ultra centrifugal filters (30 kDa MWCO) and rPfCSP was stored at −20 °C. Seventeen overlapping synthetic peptides (20 amino acids long with a 10 amino acid overlap) of Pfs25 (amino acids 18-210) were synthesized by Biomatik. Peptides were supplied as TFA salt and estimated to be >85% pure.

### Enzyme-linked immunosorbent assay (ELISA)

PfCSP and Pfs25-specific antibodies were analyzed by ELISA using Nunc MaxiSorp 96 well plates coated with 100 ng/ml rPfCSP in DPBS overnight at 4 °C, and Immulon 4hbx 96 well plates coated with 1 μg/ml rPfs25 in 0.1 M carbonate-bicarbonate buffer (pH 9.6), respectively. Pfs25-coated plates were blocked with 1% BSA and 0.1% Tween-20 in DPBS, while PfCSP-coated plates were blocked with 1% BSA in DPBS. For endpoint titer analysis, sera, in duplicate, were diluted in corresponding blocking buffers and incubated for 1 h at 25 °C. Plates were washed; first with DPBS containing 0.5% Tween-20 followed by DPBS alone. Plates were incubated with 1:2000 dilution of peroxidase-conjugated goat anti-mouse IgG (SeraCare) secondary antibody for 1 h at 25 °C, washed as above, and developed using a one-component ABTS substrate (SeraCare). After development, the enzyme reaction was stopped with 1% SDS and plates were read at 405 nm by VersaMax ELISA reader (Molecular Devices). Endpoint titers were determined using the average plus three standard deviations of the optical densities (OD) of pooled pre-immune mouse sera replicates as a cutoff. Antigen-specific antibody isotypes were evaluated for each group’s pooled serum at a dilution corresponding to the absorbance values within the linear range of the ELISA reaction, in duplicates. Peroxidase labeled goat anti-mouse IgG1, IgG2a, IgG2b, and IgG3 (Southern Biotech) were used at 1:5000 dilution, and the plates were processed as above. Antibody-antigen avidity was evaluated using sera at fixed dilutions corresponding to the absorbance values within the linear range of the ELISA reaction in duplicates. Sera were incubated at 37 °C for 1 h followed by incubation with various concentrations of NaSCN (0, 1, 2, 4, 8 M) for 15 min. Washed plates were incubated with peroxidase-labeled goat anti-mouse IgG for 1 h at 37 °C and developed as above. Avidity index was defined as the NaSCN concentration resulting in 50% dissociation of antibody-antigen binding.

### Production of fluorescently-labeled proteins

Recombinant PfCSP and Pfs25 protein were fluorescently labeled^28^. Briefly, PfCSP and Pfs25 were independently conjugated to either PE or AlexaFluor 647 using the Lightning-Link R-Phycoerythrin (R-PE) and Lightning-Link I Rapid Alexa Fluor 647 according to the manufacturer’s instructions (Novus Biologicals). Adjustment to the molar ratio was performed as per manufacturer recommendations.

### Flow cytometry analysis of memory B and GC B cells

Spleens were collected and processed using 40 µm cell strainers in complete DMEM to obtain single-cell suspensions. All steps were carried out at 4 °C. RBCs were first lysed with ACK (5 min), then splenocytes were washed twice and incubated with fluorescently labeled PfCSP or Pfs25. All cells were stained for live-dead, incubated with Fc block (Biolegend) for 20 min at 4 °C, washed with FACS buffer (1% BSA in PBS), and stained for 1 h using the antibody panel in Supplementary Table 1. Following staining, cells were washed twice, fixed with 300 µL (1% paraformaldehyde) and samples were acquired on a BD LSR II equipped with 4 laser lines and 18 PMTs. The gating strategy is provided in Supplementary Fig. 1.

### Flow cytometry analysis of T cells

Splenocytes were collected, counted using a Vi-Cell automated cell counter, resuspended at 20,000 cells/μl, and seeded into a 5 mL polypropylene FACS tube (100 μl/tube). For Pfs25 restimulation, cells were stimulated with 80 μl of a pool of 17 Pfs25 synthetic peptides at a final concentration of 2.5 μg/mL, and 1 μg/mL of CD28/CD49d mixture (co-stimulatory signal) for 6 hours. One hour post-stimulation a volume of 20 μl containing brefeldin-A and monensin at final concentrations of 0.2 and 0.14 µM, respectively, was added to the cells to block cytokine secretion. Cells were then washed using PBS, stained for live-dead cells using the Aqua LD stain (Thermo Fisher), and blocked for 20 minutes in the dark using Fc block (Biolegend) before staining with specific antibodies. Splenocytes were stained as per antibody panel in Supplementary Table 2. Samples were acquired on a BD LSR II equipped with 4 laser lines and 18 PMTs. The gating strategy is provided in Supplementary Fig. 2.

### Statistical analysis

All statistical analyses were performed using GraphPad Prism software. Two-sided Mann-Whitney U test was used to analyze antibody titers and SMFA data. The log-rank (Mantel-Cox) test was used to analyze survival curves. One way ANOVA was used to analyze avidity data. All statistical tests were conducted using a 5% significance level.

### Reporting summary

Further information on research design is available in the Nature Research Reporting Summary linked to this article.

## Supplementary information

Supplemental Material

REPORTING SUMMARY

## Data Availability

All data associated with this study are present in the paper or the Supplementary Materials. Request for resources, data and reagents should be directed to the lead contact, Nirbhay Kumar (nkumar@gwu.edu). All unique reagents described in this study are available upon request to the lead author with a completed Materials Transfer Agreement.
