# Author Correction: mRNA-LNP expressing PfCSP and Pfs25 vaccine candidates targeting infection and transmission of *Plasmodium falciparum*

**DOI:** 10.1038/s41541-023-00723-w

**Published:** 2023-08-11

**Authors:** Clifford T. H. Hayashi, Yi Cao, Leor C. Clark, Abhai K. Tripathi, Fidel Zavala, Garima Dwivedi, James Knox, Mohamad-Gabriel Alameh, Paulo J. C. Lin, Ying K. Tam, Drew Weissman, Nirbhay Kumar

**Affiliations:** 1 Department of Global Health, Milken Institute School of Public Health, George Washington University, Washington, DC 20052 USA; 2 Johns Hopkins Malaria Research Institute, Department of Molecular Microbiology and Immunology, Bloomberg School of Public Health, Johns Hopkins University, Baltimore, MD 21215 USA; 3 Department of Medicine, University of Pennsylvania, Philadelphia, PA 19104 USA; 4 Department of Pathology, University of Pennsylvania, Philadelphia, PA 19104 USA; 5 Acuitas Therapeutics, Vancouver, BC Canada

**Keywords:** RNA vaccines, Malaria

Correction to: *npj Vaccines* 10.1038/s41541-022-00577-8, published online 01 December 2022

In the Acknowledgements section of this article the grant number was incorrectly given as AI47089 and should have been AI127544. The original article has been corrected.

