# Supplementary material for: mRNA-LNP expressing PfCSP and Pfs25 vaccine candidates targeting infection and transmission of Plasmodium falciparum
Source: NPJ Vaccines. 2022 Dec 1;7:155. doi: 10.1038/s41541-022-00577-8 (PMC9715627; doi:10.1038/s41541-022-00577-8)
Supplement: Supplementary file 1 — Supplemental Material [file 41541_2022_577_MOESM1_ESM.pdf]

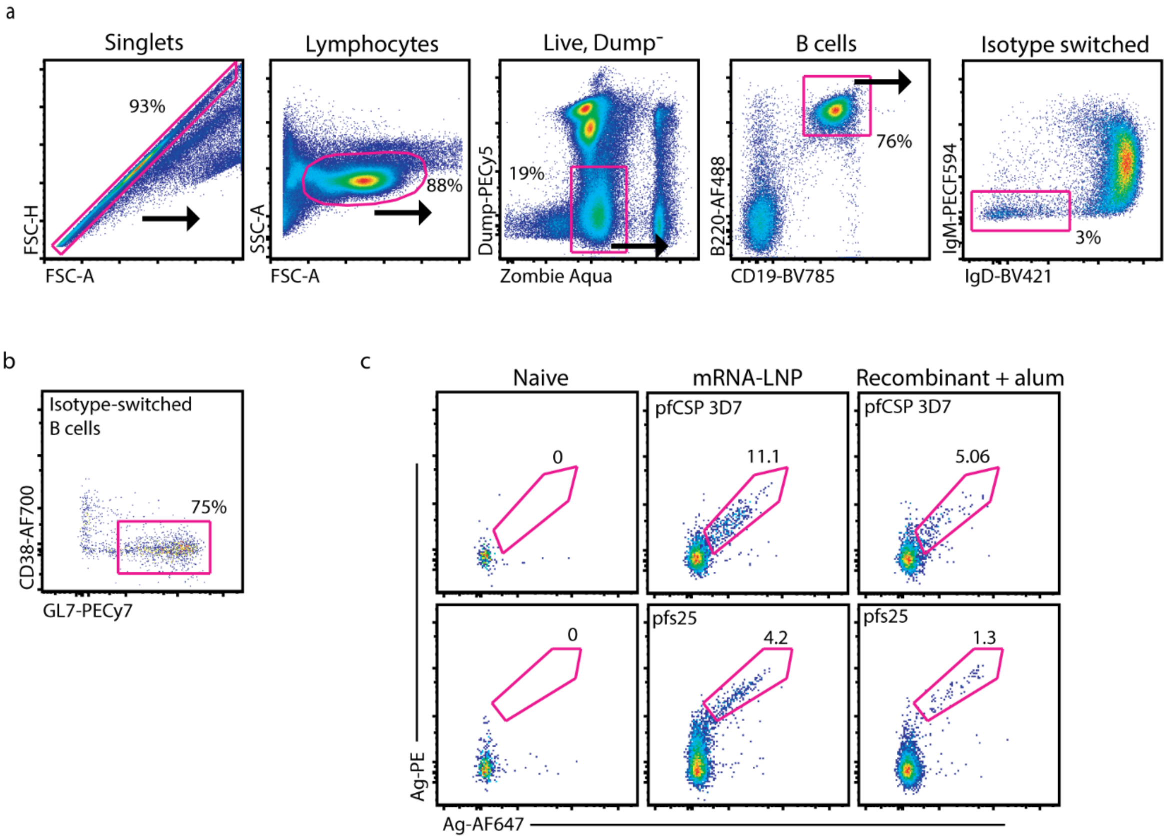

Supplementary Figure 1. B cell gating strategy. a Gating scheme to identify isotype-switched (IgD-IgM-) B cells. **b** Identification of germinal center B cells (CD38-GL7+). **c** Identification of Ag-binding B cells using fluorophore-conjugated protein probes

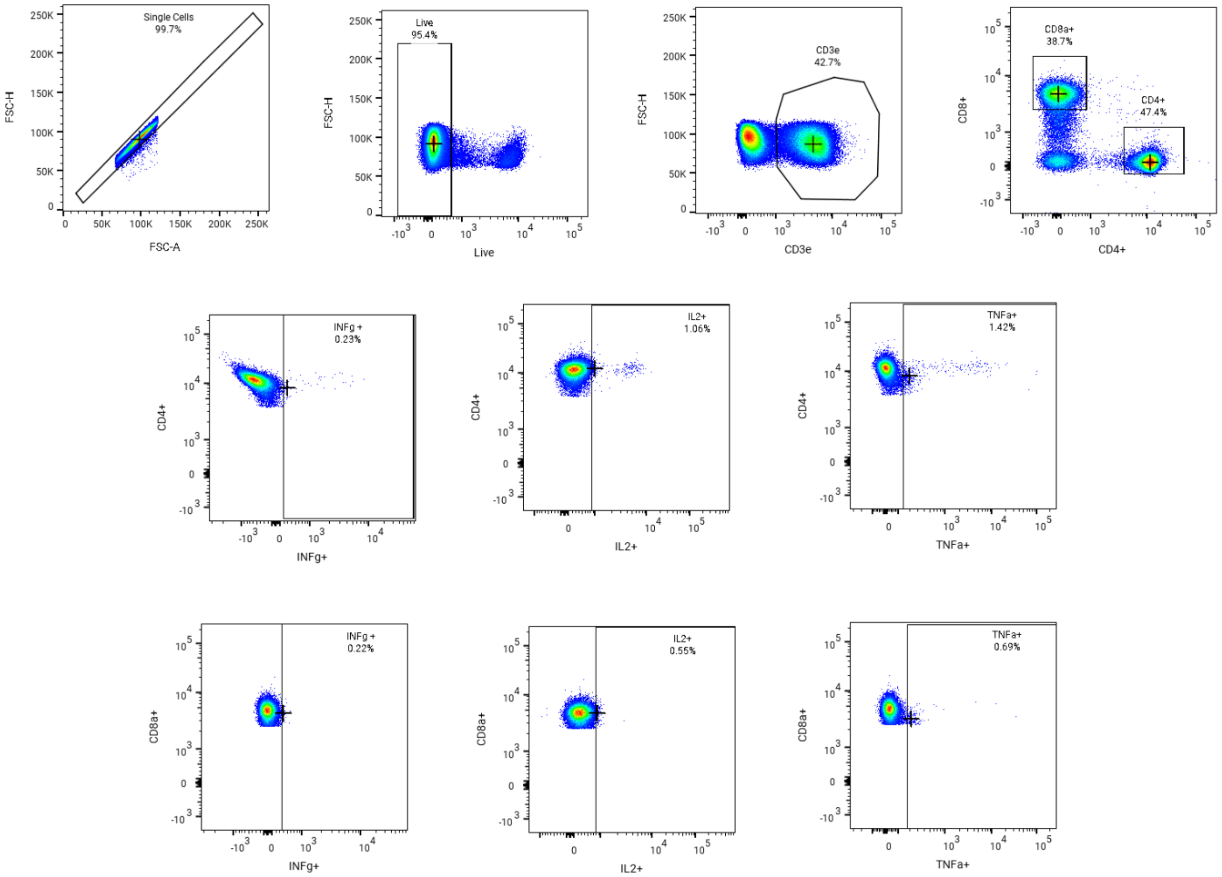

Supplementary Figure 2. T cell gating strategy. Gating scheme to identify cytokine-producing CD4 and CD8 T cells

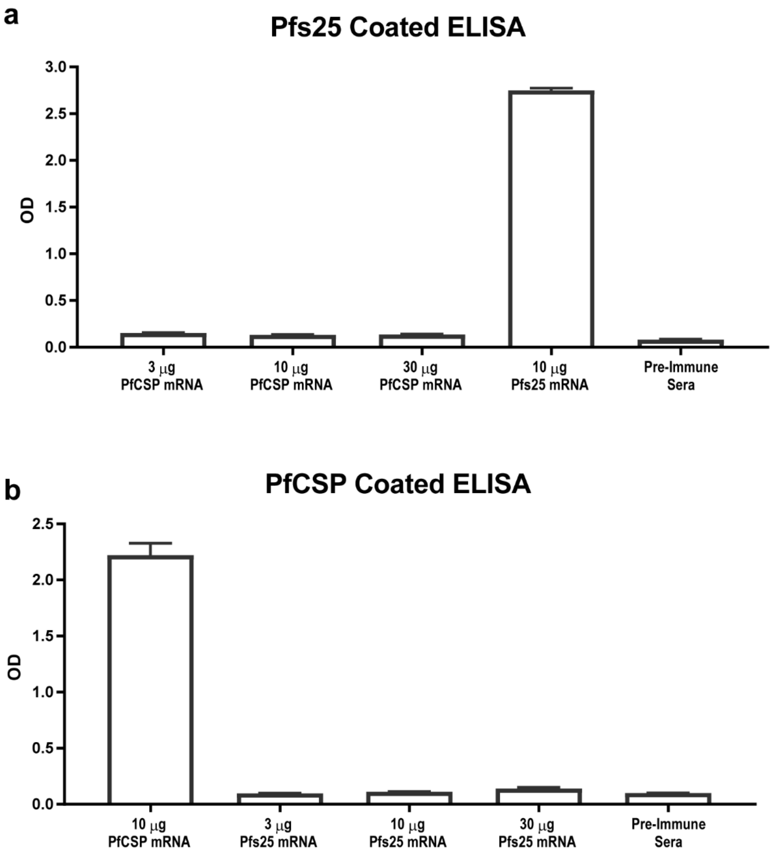

Supplementary Figure 3. Cross-reactivity of antibody responses between Pfs25 and PfCSP antigens. Sera collected from the bleed, B3 (Fig 1a), were analyzed by ELISA to determine PfCSP reactivity of Pfs25 mRNA-LNP immunized mice sera and vice-versa. Pre-immune sera were used as a negative control. Immune and pre-immune sera were tested at a fixed 1:500 dilution. Reactivity of sera to Pfs25 and PfCSP is shown in panels **a** and **b** , respectively. Error bars represent the standard deviation.

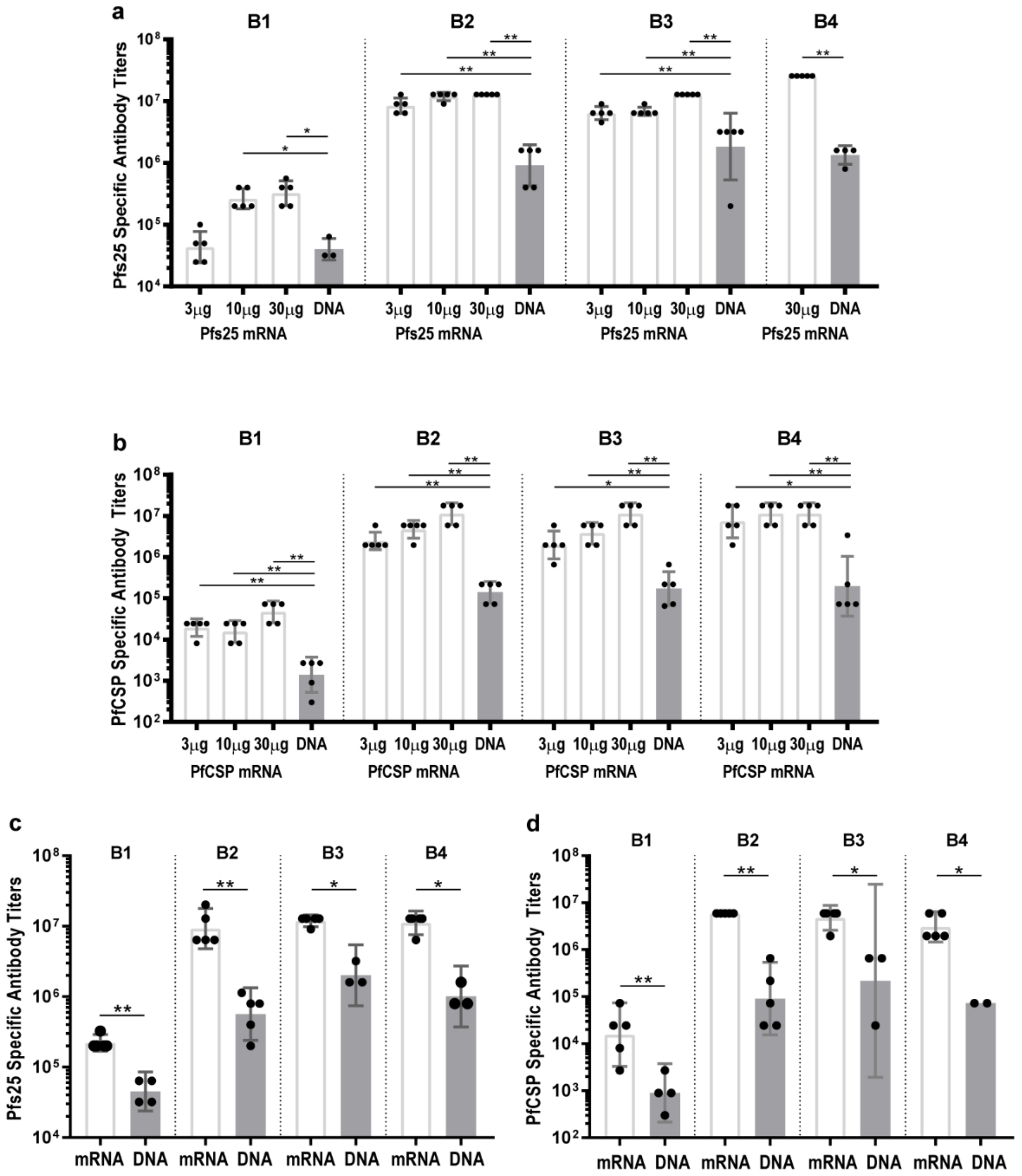

Supplementary Figure 4. Comparison of antibody responses in mice immunized with Pfs25 mRNA-LNPs, PfCSP mRNA-LNP and DNA plasmids encoding Pfs25 and PfCSP. Immunization and blood collection schedules are shown in Fig. 1a. Sera from bleeds at indicated time points were analyzed using ELISA to determine antigen-specific antibody titers reported as the geometric mean endpoint titers with 95% confidence intervals. Panel **a** reports Pfs25-specific antibody titers of mice immunized with Pfs25 immunogens (N=5 for all groups except DNA group at time point B1, N=3 and at B4, N=4). panel **b** reports PfCSP-specific titers of mice immunized with PfCSP immunogens (N=5 for all groups). Panel **c** and **d** report Pfs25-specific and PfCSP-specific antibody titers of mice co-immunized with Pfs25 and

PfCSP immunogens (N=5 for all groups except for DNA at time point B1, N=4, DNA at time points B3 and B4, N=3). ELISA data for mRNA immunization groups are exactly the same as in Fig. 2 and 3, however, shown here for comparison with DNA immunization groups (shaded grey). Statistical analysis was performed using a two-sided Mann-Whitney U test (*$p<0.05$; **$p<0.01$).

Supplementary Table 1. A detailed list of antibodies used for B cell analysis by Flow Cytometry

Marker	Clone	Dilution	Catalog Numbers	Source
B220	RaA3-6B2	1:200	103225	Biolegend
IgD	11-26c.2a	1:300	405725	Biolegend
Zombie Aqua	n/a	1:400	423102	Biolegend
CD19	6D5	1:100	115543	Biolegend
CD38	90	1:100	56-0381-82	Invitrogen
IgM	R6-60.2	1:200	562565	BD Bioscience
CD4	H129.19	1:800	130312	Biolegend
CD8	53-6.7	1:400	553034	BD Bioscience
Gr-1	RB6-8C5	1:800	108410	Biolegend
F4/80	BM8	1:800	15-4801-82	Invitrogen
GL7	GL7	1:200	144620	Biolegend

Supplementary Table 2. A detailed list of antibodies used for T cell analysis by Flow Cytometry

Marker	Clone	Dilution	catalog numbers	Source
CD107a	1D4B	1:100	553793	BD Bioscience
CD4	GK1.5	1:200	100434	Biolegend
CD8a	53-6.7	1:200	100725	Biolegend
Live/Dead		1:200	L34966	Invitrogen
CD3e	145-2C11	1:200	100351	Biolegend
IL-4	11B11	1:200	564006	BD Bioscience
IL-2		1:200	554429	BD Bioscience
IFN-g	XMG1.2	1:200	557998	BD Bioscience
IL-17	TC11-18H10.1	1:200	506940	BD Bioscience
IL-5	TRFK5	1:200	504304	BD Bioscience
TNF-a	MP6-XT22	1:200	557644	BD Bioscience
